# Association of age-adjusted Charlson comorbidity index with adverse outcomes in patients undergoing transcatheter aortic valve replacement: A retrospective cohort study

**DOI:** 10.1097/MD.0000000000036283

**Published:** 2023-11-24

**Authors:** Hua Yang, Limin Meng, Shuanli Xin, Chao Chang, Xiufeng Zhao, Bingyan Guo

**Affiliations:** a Department of Cardiology, The Second Hospital of Hebei Medical University, Shijiazhuang, Hebei, China; b Department of Cardiology, Handan First Hospital, Handan, Hebei, China.

**Keywords:** age-adjusted Charlson comorbidity index, all-cause mortality, readmission, transcatheter aortic valve replacement

## Abstract

Patients undergoing transcatheter aortic valve replacement (TAVR) have a high comorbid burden. Our objective was to assess the association between the age-adjusted Charlson comorbidity index (Age-CCI) and mortality and readmission rates within 1-year post-TAVR. Data were extracted from the Medical Information Mart for Intensive Care IV database (MIMIC-IV version 2.0). The primary endpoint was a composite outcome of all-cause mortality or readmission within 1-year after TAVR. To examine the associations of Age-CCI with outcomes, we used multivariable Cox proportional hazards regression, restricted cubic spline (RCS), and Kaplan–Meier curves. A total of 785 patients (52.9% male) with a median age of 84.0 years were assessed. More than half of our patients had an Age-CCI ≥ 7. After adjustment for potential confounders, we found that a 1 unit increase in Age-CCI was associated with a 10% increase in mortality and readmission rates after TAVR (HR = 1.10, 95% CI: 1.04–1.17, *P* < .001). High Age-CCI (Age-CCI ≥ 7) compared with the low Age-CCI (Age-CCI < 7) showed a 36% increase of mortality and readmission rates (HR = 1.36, 95% CI: 1.07–1.73, *P =* .013). The RCS curve analysis showed a continuous linear relationship between Age-CCI and the composite outcome risk (*P* for non-linearity = .671). The Kaplan–Meier survival analysis showed that patients with Age-CCI ≥ 7 had a poorer prognosis than those with Age-CCI < 7 (log-rank *P* < .001). Subgroup analyses showed the results remained stable. Age-CCI is independently associated with all-cause mortality and readmission in patients treated with TAVR, which may help clinicians risk-stratify patients and offer an opportunity to improve patient outcomes.

## 1. Introduction

With the rapid advancement of device technology, transcatheter aortic valve replacement (TAVR) is now indicated for low- and intermediate-risk patients, and it is increasingly becoming the main method for treating patients with aortic stenosis.^[1–3]^ In addition, it has been confirmed that TAVR is feasible for people with rheumatic aortic stenosis and pure aortic regurgitation.^[4,5]^ So, the number of candidates for TAVR will further increase. Despite numerous improvements in TAVR procedures and expert experience, the rates of death or readmission after TAVR remain very high, with 1-year mortality rates of approximately 24% in high-risk patients and readmission rates ranging from 18.4% to 59.7%.^[6–8]^ Moreover, the economic burden of health care is significantly increased by readmission and mortality.^[9,10]^ Identifying high-risk populations is critical to making timely individualized interventions and maintaining TAVR cost-effective.

The majority of TAVR patients have a significant burden of comorbidities. Furthermore, approximately half of the causes of death or readmission following TAVR are non-cardiac and related to baseline comorbidities.^[11,12]^ Therefore, cardiac teams need to assess chronic disease burden in the decision-making process for TAVR eligibility. The Charlson Comorbidity Index (CCI) is an indicator that quantifies comorbidity burden by aggregating individually weighted scores for 19 comorbidities. The Age-adjusted Charlson comorbidity index (Age-CCI), which incorporates age and comorbidities, outperformed CCI in terms of discriminating and predictive accuracy.^[13,14]^

The effect of CCI on outcomes following TAVR is still controversial. Higher CCI was independently linked to higher mortality for up to 6 years after TAVR in 1 study.^[15]^ However, Hiltrop et al found no difference in CCI scores between survivors and deceased populations at 2 years follow-up post-TAVR.^[16]^ Another study also found that the CCI score was not independently associated with a higher risk of 30-day death but can be useful in addition to Logistic Euro Score (LES) and Society of Thoracic Surgeons (STS) risk models in informing decision making on the selection of patients for TAVR.^[17]^ Furthermore, it is unknown whether Age-CCI is associated with 1-year readmission rate in patients who underwent TAVR. Therefore, the goal of this study was to evaluate the burden of comorbidities in patients receiving TAVR, as well as the association of Age-CCI with adverse outcomes within 1-year after TAVR.

## 2. Materials and Methods

### 2.1. Data source

The Medical Information Mart for Intensive Care IV (MIMIC-IV version 2.0) provided the information used in this study. The database was publicly available of patients hospitalized at the Beth Israel Deaconess Medical Center between 2008 and 2019.^[18]^ We passed the exam named “Protecting Human Research Participants” and obtain the seniority to access these database (Record ID:48851221). The study complied with the principles of the Declaration of Helsinki. The institutional review boards of the Massachusetts Institute of Technology and Beth Israel Deaconess Medical Center approved the study, and informed consents were exempted due to all patients’ data were anonymized before the data were obtained. We present this article by the Strengthening the Reporting of Observational Studies in Epidemiology (STROBE) guidelines.

### 2.2. Study population

We identified patients who underwent endovascular TAVR using International Classification of Diseases, Tenth Revision, Clinical Modification procedural codes (02RF37Z, 02RF38Z, 02RF3JZ, 02RF3KZ, or X2RF332) and ICD-9-CM procedural codes of 35.05. If patients underwent repeat TAVR, only patients with the first procedure were included.

### 2.3. Study variables

Age, gender, previous cardiac surgery, biomarkers, and associated comorbidities were extracted. Identification of disease components in CCI based on ICD-9 and ICD-10 diagnosis codes included myocardial infarction, congestive heart disease, peripheral vascular disease, cerebrovascular disease, dementia, chronic pulmonary disease, rheumatic disease, peptic ulcer disease, liver disease, diabetes with or without complications, paraplegia, renal disease, malignant tumors, and Acquired Immune Deficiency Syndrome (AIDS). Additionally, we analyzed conditions such as coronary artery disease, hypertension, atrial fibrillation/flutter (AF), pulmonary hypertension, anemia, and obesity that were not part of the CCI. Other variables were extracted including previous cardiac procedures, in-hospital procedures such as percutaneous coronary intervention, previous coronary artery bypass grafting, in-hospital complications, such as peri-valve leakage, conversion to surgical aortic valve replacement, acute kidney injury (AKI), blood transfusion, permanent pacemaker placement, transient ischemic attack/stroke, cardiogenic shock, and cardiac arrest.

### 2.4. Comorbidity evaluation

The calculated Age-CCI for each patient was used to measure the comorbidity burden. A total of 19 disease components of CCI were identified. Score weights for diseases used the original weights from Charlson (Supplemental Table 1, http://links.lww.com/MD/K823). The age-based scoring was 0 points for ages under 40, 1 point for each additional 10 years between 40 and 70, and 4 points for ages 70 and older. The final score is the sum of the comorbidity score and the age group score that corresponds. The burden of comorbidities increases with the score. This score divided patients into 2 categories based on their median: low (Age-CCI < 7) and high Age-CCI (Age-CCI ≥ 7).

### 2.5. Outcomes

Our primary outcome was the combined result of 1-year all-cause mortality and readmission. Readmission was defined as any unplanned hospital readmission for any reason within the first year after discharge. Only the first readmission was included for patients who had several readmissions. Time to readmission was calculated from the time of discharge to the time of the first readmission. The time to the composite outcome was the time of the first occurrence of any such event.

### 2.6. Statistical analysis

Continuous variables were provided as mean ± SD or median and interquartile range (IQR) depending on their distribution, as determined by the Shapiro–Wilk test, and were compared using t tests or the Mann–Whitney U test as appropriate. Chi-square tests or the Fisher exact test were used to compare categorical data reported as numbers and percentages. Missing data were imputed with the corresponding median or mean value.

A multivariable Cox proportional hazards model was used to assess the independent association between Age-CCI and the primary outcome, after testing the proportional hazards assumption. Results were presented as hazard ratios (HR) with 95% confidence intervals (CI). Variables with *P* < .05 in univariable analysis were selected as adjustments that were recorded in the dataset and were not part of the CCI, nor were they in the causal chain between CCI and outcome. Model 1: unadjusted. Model 2: adjusted for age and gender. Model 3 adjusted for Model 2 plus red blood cell, mean corpuscular hemoglobin concentration, red blood cell distribution width, creatinine, chloride, hypertension, and atrial fibrillation/flutter. The variance inflation factor (VIF) was applied to examine the collinearity of the variables (acceptable collinearity VIF ≤ 5).

Kaplan–Meier survival curves were generated, and the log-rank test was utilized to compare the groups. A restricted cubic spline (RCS) was used to examine the functional relationship between Age-CCI and outcome. Additionally, we conducted subgroup analyses by age (< 84 and ≥ 84 years, median), gender, anemia (hemoglobin men < 13 g/dL, women < 12 g/dL), hypertension, atrial fibrillation/flutter, and renal disease. The continuous variable was transformed into a categorical variable using the clinical cut points or median value. The interaction was tested via a likelihood ratio test.

All statistical analyses were performed using R software (version 3.6.2; R Foundation for Statistical Computing, Vienna, Austria) and Stata statistical software (version 14; Stata Corp LP, College Station, TX). A 2-sided *P* < .05 was considered statistically significant.

## 3. Results

### 3.1. Baseline characteristics

**Figure 1.**
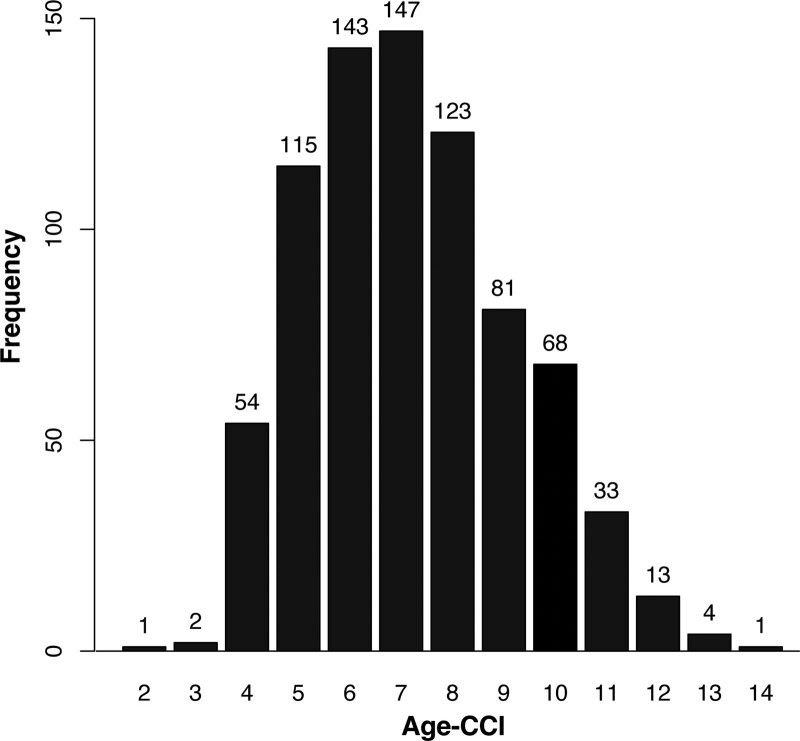
Distribution of total Age-CCI. Age-CCI = age-adjusted Charlson comorbidity index.

The current study included 785 patients in total (Supplemental Fig. 1, http://links.lww.com/MD/K821). Table 1 presents the patient baseline characteristics of the entire cohort and different Age-CCI groups. The median age was 84.0 years old, ranging from 51 to 98 years, and 52.9% of them were male. The average Age-CCI was 7.2, with a minimum of 2 and a maximum of 14 (Fig. 1).

**Table 1:** Baseline characteristics of participants.

Variables	Total (n = 785)	Age-CCI < 7 (n = 315)	Age-CCI ≥ 7 (n = 470)	*P* value
Age (yr)	84.0 (77.0, 88.0)	84.0 (77.0, 88.0)	83.0 (77.0, 88.0)	.669
Male, n (%)	416 (53.0)	149 (47.3)	267 (56.8)	.009
BMI (kg/m^2^)	28.2 ± 6.9	27.6 ± 6.9	28.± 6.9	.072
Age-CCI	7.2 ± 2.0	5.3 ± 0.8	8.5 ± 1.5	<.001
Age-CCI component				
Age-score, n (%)				.071
2	17 (2.2)	11 (3.5)	6 (1.3)	
3	57 (7.3)	26 (8.3)	31 (6.6)	
4	711 (90.6)	278 (88.3)	433 (92.1)	
Myocardial infarction, n (%)	163 (20.8)	30 (9.5)	133 (28.3)	<.001
Congestive heart failure, n (%)	559 (71.2)	159 (50.5)	400 (85.1)	<.001
Peripheral vascular disease, n (%)	161 (20.5)	32 (10.2)	129 (27.4)	<.001
Cerebrovascular disease, n (%)	107 (13.6)	23 (7.3)	84 (17.9)	<.001
Dementia, n (%)	27 (3.4)	7 (2.2)	20 (4.3)	.126
Chronic pulmonary disease, n (%)	263 (33.5)	67 (21.3)	196 (41.7)	<.001
Rheumatic disease, n (%)	62 (7.9)	20 (6.3)	42 (8.9)	.188
Peptic ulcer disease, n (%)	3 (0.4)	0 (0)	3 (0.6)	.278
Mild liver disease, n (%)	40 (5.1)	5 (1.6)	35 (7.4)	<.001
Diabetes				
Without complications, n (%)	159 (20.3)	53 (16.8)	106 (22.6)	.050
With complications, n (%)	139 (17.7)	3 (1)	136 (28.9)	<.001
Paraplegia, n (%)	16 (2.0)	1 (0.3)	15 (3.2)	.005
Renal disease, n (%)	300 (38.2)	17 (5.4)	283 (60.2)	<.001
Malignant cancer, n (%)	51 (6.5)	2 (0.6)	49 (10.4)	<.001
Moderate or severe liver disease, n (%)	15 (1.9)	1 (0.3)	14 (3)	.008
Metastatic solid tumor, n (%)	7 (0.9)	0 (0)	7 (1.5)	.046
AIDS, n (%)	1 (0.1)	0 (0)	1 (0.2)	1
Other co-morbidities				
Pulmonary hypertension, n (%)	81 (10.3)	24 (7.6)	57 (12.1)	.042
Coronary artery disease, n (%)	476 (60.6)	165 (52.4)	311 (66.2)	<.001
Hypertension, n (%)	271 (34.5)	185 (58.7)	86 (18.3)	<.001
Atrial fibrillation/flutter, n (%)	368 (46.9)	127 (40.3)	241 (51.3)	.003
Anemia, n (%)	620 (79.0)	223 (70.8)	397 (84.5)	<.001
Obesity, n (%)	124 (15.8)	42 (13.3)	82 (17.4)	.121
Prior PCI, n (%)	193 (24.6)	60 (19)	133 (28.3)	.003
Prior CABG, n (%)	119 (15.2)	44 (14)	75 (16)	.446
Prior PPM, n (%)	82 (10.4)	37 (11.7)	45 (9.6)	.093
Baseline biomarkers				
RBC (10^12^/L)	3.7 ± 0.6	3.8 ± 0.6	3.6 ± 0.6	<.001
WBC (10^9^/L)	7.0 (5.5, 8.6)	6.9 (5.5, 8.6)	7.1 (5.6, 8.6)	.674
Platelet (10^9^/L)	183.0 (144.0, 226.0)	181.5 (147.0, 225.8)	184.0 (143.0, 227.0)	.644
Hematocrit (%)	34.1 ± 5.3	35.3 ± 4.8	33.3 ± 5.5	<.001
Hemoglobin (g/dL)	11.0 ± 1.9	11.5 ± 1.7	10.7 ± 1.9	<.001
MCH (pg)	29.8 ± 2.6	30.1 ± 2.4	29.6 ± 2.7	.014
MCHC (g/dL)	32.2 ± 1.5	32.5 ± 1.4	32.0 ± 1.5	<.001
MCV (fL)	92.6 ± 6.5	92.6 ± 5.9	92.7 ± 6.8	.827
RDW (%)	14.6 (13.6, 15.9)	14.0 (13.2, 15.2)	15.0 (13.9, 16.2)	<.001
Anion gap (mEq/L)	14.2 ± 2.9	13.8 ± 2.8	14.6 ± 3.0	<.001
Bicarbonate (mEq/L)	25.7 ± 3.8	25.7 ± 3.4	25.7 ± 4.0	.964
BUN (mg/dL)	27.9 ± 15.2	22.3 ± 9.2	31.7 ± 17.2	<.001
Chloride (mEq/L)	102.0 (99.0, 105.0)	102.0 (100.0, 105.0)	102.0 (99.0, 105.0)	.212
Creatinine (mg/dL)	1.3 ± 0.9	1.0 ± 0.5	1.5 ± 1.1	<.001
Sodium (mEq/L)	140.0 (137.0, 141.0)	140.0 (137.0, 142.0)	140.0 (137.0, 141.0)	.856
Potassium (mEq/L)	4.2 (3.9, 4.5)	4.2 (3.9, 4.5)	4.2 (3.9, 4.6)	.047
In-hospital procedures				
CABG, n (%)	20 (2.5)	13 (4.1)	7 (1.5)	.022
PCI, n (%)	109 (13.9)	38 (12.1)	71 (15.1)	.227
In-hospital complications				
Leakage, n (%)	14 (1.8)	5 (1.6)	9 (1.9)	.734
SAVR, n (%)	19 (2.4)	8 (2.5)	11 (2.3)	.859
Blood transfusion, n (%)	155 (19.7)	44 (14)	111 (23.6)	<.001
PPM, n (%)	180 (22.9)	66 (21)	114 (24.3)	.281
AKI, n (%)	148 (18.9)	30 (9.5)	118 (25.1)	<.001
Cardiac arrest, n (%)	27 (3.4)	8 (2.5)	19 (4)	.257
Cardiogenic shock, n (%)	28 (3.6)	5 (1.6)	23 (4.9)	.014
TIA stroke, n (%)	40 (5.1)	8 (2.5)	32 (6.8)	.008
Outcomes				
1-yr mortality, n (%)	131 (16.7)	25 (7.9)	106 (22.6)	<.001
Mortality and readmission, n (%)	352 (44.8)	114 (36.2)	238 (50.6)	<.001
Length of hospital (d)	5.0 (3.0, 9.0)	4.0 (3.0, 7.0)	6.0 (4.0, 10.0)	<.001

Age-CCI = age-adjusted Charlson comorbidity index, AIDS = Acquired Immune Deficiency Syndrome, AKI = acute kidney injury, BMI = body mass index, BUN = blood urea nitrogen, CABG = coronary artery bypass grafting, MCV = mean corpuscular volume, MCH = mean corpuscular hemoglobin, MCHC = mean corpuscular hemoglobin concentration, PCI = percutaneous coronary intervention, PPM = permanent pacemaker, RDW = red blood cell distribution width, RBC = red blood cells, SAVR = surgery aortic valve replacement, WBC = white blood cells.

Although the age of the patients did not significantly differ across groups, more male patients were found in the high Age-CCI groups. The high Age-CCI groups had significantly higher rates of myocardial infarction, congestive heart failure, cerebrovascular disease, chronic pulmonary disease, diabetes, peripheral vascular disease, liver disease, renal disease, paraplegia, coronary artery disease, anemia, atrial fibrillation/flutter, hypertension, and cancer (*P* < .01); however, the prevalence of the other comorbidities did not differ significantly.

The most common cardiovascular comorbidities were congestive heart failure (559 patients, 71.2%) and coronary artery disease (476 patients, 60.6%), while the most common non-cardiovascular comorbidities were anemia (620 patients, 79.0%), renal disease (300 patients, 38.2%), diabetes (298 patients, 38.0%), and chronic pulmonary disease (263 patients, 33.5%).

### 3.2. Age-CCI and primary outcomes

Data on 1-year mortality or readmission rates were available for 352 (44.8%) patients, of which 131 (16.7%) died within 1-year after discharge. Table 2 presents the association between Age-CCI and outcome. In the univariable analysis, Age-CCI was found to be significantly associated with the composite of 1-year death or readmission (HR = 1.14, 95% CI:1.09–1.20, *P <* .001). In multivariable adjustment analysis (Model 3), the association remained statically significant (HR = 1.10, 95% CI: 1.04–1.17, *P* < .001), showing that with every 1-unit increase in the Age-CCI, the risk of primary outcome increases by 10%. Similarly, the multivariate-adjusted risk of 1-year death increased significantly with a unit increase in Age-CCI (HR = 1.21, 95% CI:1.10–1.33, *P <* .001).

**Table 2:** Cox regression analysis of the association between Age-CCI and outcomes.

Variables	Model 1		Model 2		Model 3	
	HR (95% CI)	*P* value	HR (95% CI)	*P* value	HR (95% CI)	*P* value
Mortality and readmission						
Age-CCI	1.14 (1.09~1.20)	<.001	1.14 (1.09~1.20)	<.001	1.10 (1.04~1.17)	.001
Age-CCI groups						
Age-CCI < 7	Reference		Reference		Reference	
Age-CCI ≥ 7	1.63 (1.30~2.03)	<.001	1.61 (1.29~2.02)	<.001	1.36 (1.07~1.73)	.013
1-yr mortality						
Age-CCI	1.31 (1.21~1.42)	<.001	1.31 (1.21~1.42)	<.001	1.21 (1.10~1.33)	<.001
Age-CCI groups						
Age-CCI < 7	Reference		Reference		Reference	
Age-CCI ≥ 7	3.11 (2.01~4.81)	<.001	2.96 (1.91~4.59)	<.001	1.96 (1.22~3.15)	.005

Model 1 adjusted for nothing.

Model 2 adjusted for age and gender.

Model 3 adjusted for age, gender, red blood cell, mean corpuscular hemoglobin concentration, red blood cell distribution width, creatinine, chloride, hypertension, and atrial fibrillation/flutter.

Age-CCI = age-adjusted Charlson comorbidity index, HR = hazard ratio, CI = confidence interval.

**Figure 2.**
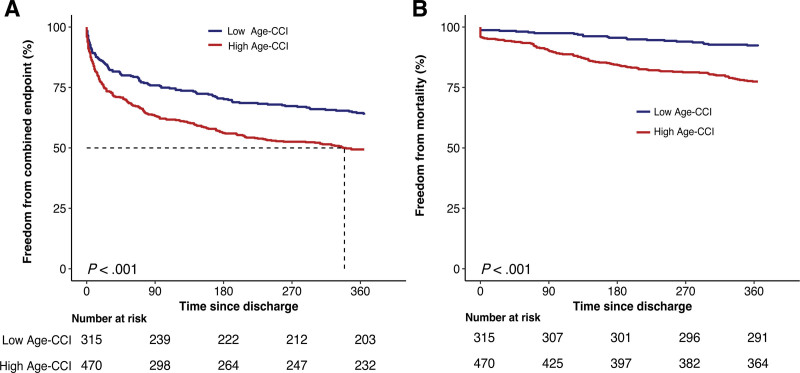
Kaplan–Meier curves for the combined endpoint (A) and all-cause mortality (B). Age-CCI = age-adjusted Charlson comorbidity index.

We also transformed the Age-CCI from a continuous variable to a dichotomous variable for sensitivity analysis. An Age-CCI score ≥ 7 was associated with a higher risk of the primary composite endpoint (HR = 1.36, 95% CI: 1.07–1.73, *P* = .013) and 1-year mortality (HR = 1.96, 95% CI:1.22–3.15, *P* = .005) after adjusting for all the variables in Model 3. Figure 2 depicts the Kaplan–Meier plot between the Age-CCI groups (log-rank *P <* .001).

**Figure 3.**
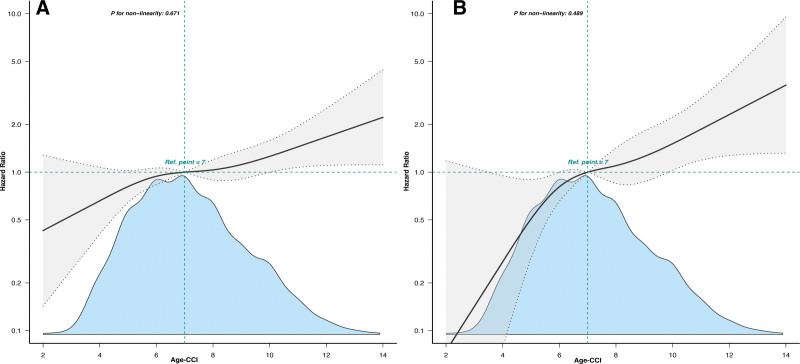
Relationship between Age-CCI and the combined endpoint (A) and all-cause mortality (B). Adjusted for all covariates in Model 3. The solid and dashed lines represent the estimated values and 95% confidence intervals. Age-CCI = age-adjusted Charlson comorbidity index.

RCS curve showed that there was a linear relationship between Age-CCI and the primary endpoint (*P* for non-linearity: 0.671, Fig. 3A). A similar result was also found in the relationship between Age-CCI and 1-year mortality (*P* for non-linearity: 0.489, Fig. 3B).

### 3.3. Subgroup analysis

**Figure 4.**
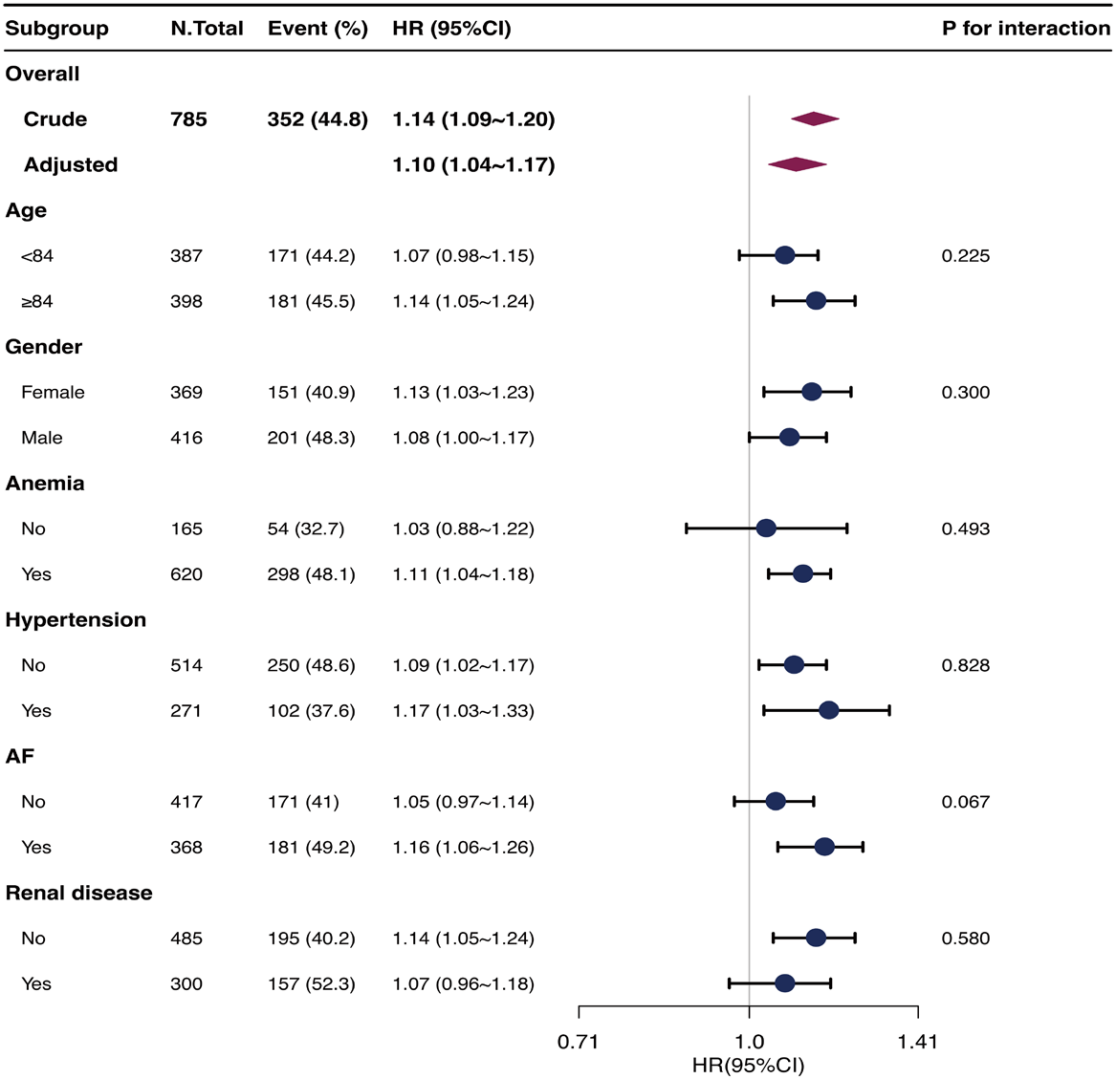
Results from subgroup analyses showing the association between Age-CCI and the primary outcome. Adjusted for age, gender (not for gender-stratified analysis), red blood cell, mean corpuscular hemoglobin concentration, red blood cell distribution width, creatinine, chloride, hypertension (not for hypertension-stratified analysis), and AF (not for AF-stratified analysis). Age-CCI = age-adjusted Charlson comorbidity index, AF = atrial fibrillation/flutter.

The risk associations between Age-CCI and the composite outcome were similar across subgroups defined by age, gender, anemia, hypertension, AF, and renal disease when Age-CCI was used as a continuous variable. No statistically significant interaction was observed (Fig. 4). The subgroup analyses produced similar results as well when mortality was an outcome (Supplemental Fig. 2, http://links.lww.com/MD/K822). Although some subgroups did not show a significant association, this trend was observed in those subgroups.

## 4. Discussion

The main finding of this study was that the Age-CCI was linearly associated not only with all-cause mortality, but also with readmission rate in TAVR populations, with the incremental risk of mortality and readmission an estimated 10% per 1-point increase in the Age-CCI score. Subgroup results suggested that this relationship was robust.

In this study, subjects with TAVR had a significant comorbidity burden, with more than half of the patients having an Age-CCI ≥ 7. This finding is in line with previous research suggesting that comorbidities are common in patients undergoing TAVR. Patients with low comorbidity burdens tend to be candidates for surgical aortic valve replacement. Previous studies have found weak associations between CCI and established preoperative risk scores such as the Society of Thoracic Surgeons (STS), Logistic Euro-SCORE (LES), and Euro-SCORE-II (ESII).^[19]^ These preoperative risk assessments did not adequately capture the risks associated with comorbidity burden and performed poorly in predicting hospitalization and 30-day mortality after TAVR.^[20]^ Therefore, it is critical to assess the comorbidity burden in TAVR patients.

In this era of newer technology providing safer procedures, we recognize that the mortality following TAVR is decreasing. Therefore, in this study, we analyzed not only deaths but also readmissions following TAVR. We found that mortality or readmission risk within 1-year following TAVR increased 10% for every 1-unit increment in the Age-CCI. Subgroup analysis suggested that the association between Age-CCI and poor outcomes was stable. Some subgroup results were not significantly correlated, possibly because of smaller sample sizes after stratification, but this trend was observed in these subgroups. Previous studies found that 30-day readmissions following TAVR were common and associated with baseline comorbidities, with more than 50% of readmissions due to noncardiac factors.^[11,12]^ This may explain the current research findings, which show that the more complications, the higher the risk of readmission and mortality. In addition, our study found that patients with high Age-CCI had a longer length of hospital stay, which may also indicate critical illness, leading to high mortality and readmission rates. AKI is frequently found in patients following TAVR and is associated with increased mortality.^[21]^ In our study, patients with high Age-CCI had a significantly greater incidence of AKI, which may lead to higher readmissions and mortality. The effect of Age-CCI on mortality and readmission in the current study emphasizes the importance of comorbidities in TAVR recipients.

In addition, our results also indicate that each 1-unit Age-CCI was associated with increased 1-year mortality. This finding is in line with previous reports.^[15,22]^ They discovered that increased mortality after 30 days was linked to greater CCI. But we should also be aware of studies with conflicting findings. Hiltrop et al analyzed 145 patients who received TAVR, of whom 61% via the femoral artery and 39% transapical, with a median [IQR] age of 84 [80–87] years and a median [IQR] CCI of 3 [2–4]. They found that CCI scores at baseline did not appear to differ between patients who died and patients who survived at 2 years of follow-up after TAVR.^[16]^ The difference in the number and characteristics of participants may be the main reason for the different results. Their patients had significantly lower comorbidities than our study population, including patients with both transfemoral and transapical approaches, whereas our study did not include patients with transapical approaches.

As in previous studies, comorbidities in the CCI component when evaluated alone were associated with poorer outcomes after TAVR. For example, chronic kidney disease, chronic lung disease, and atrial fibrillation were associated with poor long-term or short-term outcomes after TAVR.^[23–25]^ However, in the real world, TAVR recipients tend to have multiple comorbidities, and analyses of isolated chronic conditions do not adequately capture the complexity of patients. Multiple comorbidities and multiple treatments may interact with each other, raising the chance of adverse outcomes. Given the harmful impact of comorbidity, we should evaluate and manage the entire person rather than focusing on a particular disease component.

Our findings have certain clinical implications. The Age-CCI may be used as an adjunct to assess mortality or readmission rates after TAVR. An improved understanding of the burden of comorbidities may aid in preoperative risk stratification and shared decision-making. Establishing risk stratification can facilitate timely, individualized intervention, different follow-up plans, and better patient outcomes. Preoperative addressing modifiable patient comorbidity may serve as a strategy to improve postoperative outcomes and reduce cost. Further research is needed to explore the appropriate prevention and therapy of multiple comorbidities in patients with TAVR.

Our study has several limitations. First, our sample size is limited, and we only considered the variables in the database for our analysis. Information on surgical risk scores, valve type, and echocardiographic results were not available. Further research with a large sample size is needed. Second, despite performing a multivariable cox regression model to adjust for many factors, unmeasured confounders may persist, and a prospective study should be considered to confirm our findings. Third, we only used the Age-CCI to quantify the comorbidity burden. Even though this is the most extensively researched comorbidity index for predicting mortality, the results might be different if other comorbidity scales were utilized. Lastly, we cannot view the findings as causative but as an association. Future prospective studies may aid in determining the causal relationship between Age-CCI and crucial outcomes after TAVR, such as quality of life.

## 5. Conclusion

The Age-CCI was independently associated with an increased risk of readmission and mortality. This emphasizes the significance of investigating the burden of comorbidities and taking preventative measures to manage some modifiable comorbidities before surgery to reduce readmission and death following TAVR.

## Author contributions

**Conceptualization:** Bingyan Guo.

**Data Curation:** Hua Yang, Limin Meng, Chao Chang, and Xiufeng Zhao.

**Formal analysis:** Hua Yang, Limin Meng, Chao Chang, and Xiufeng Zhao.

**Investigation:** Hua Yang, Limin Meng.

**Methodology:** Shuanli Xin, Bingyan Guo.

**Supervision:** Bingyan Guo.

**Writing – original draft:** Hua Yang, Limin Meng.

**Writing – review & editing:** Shuanli Xin, Bingyan Guo.

## Supplementary Material
