# Supplementary material for: Association of age-adjusted Charlson comorbidity index with adverse outcomes in patients undergoing transcatheter aortic valve replacement: A retrospective cohort study
Source: Medicine (Baltimore). 2023 Nov 24;102(47):e36283. doi: 10.1097/MD.0000000000036283 (PMC10681598; doi:10.1097/MD.0000000000036283)
Supplement: Supplementary file 1 [file medi-102-e36283-s001.docx]

**Supplemental Table 1** Distribution of various disease weights in Age-CCI.

| **Comorbidity** | **Assigned Weights for Diseases** |
| --- | --- |
| Myocardial infarction | 1 |
| Congestive heart failure | 1 |
| Peripheral vascular disease | 1 |
| Cerebrovascular disease | 1 |
| Dementia | 1 |
| Peptic ulcer disease | 1 |
| Chronic pulmonary disease | 1 |
| Rheumatic disease | 1 |
| Diabetes with chronic complication | 2 |
| Diabetes without chronic complication | 1 |
| Moderate or severe renal disease | 2 |
| Paraplegia | 2 |
| Mild liver disease | 1 |
| Moderate or severe liver disease | 3 |
| Any malignancy, including lymphoma and leukemia | 2 |
| Metastatic solid tumor | 6 |
| AIDS | 6 |
| Age-score |  |
| 40-50 | 1 |
| 51-60 | 2 |
| 61-70 | 3 |
| >70 | 4 |

Age-CCI = age-adjusted Charlson comorbidity index, AIDS = Acquired Immune Deficiency Syndrome.
