# Supplementary figures and images for: Association of age-adjusted Charlson comorbidity index with adverse outcomes in patients undergoing transcatheter aortic valve replacement: A retrospective cohort study
Source: Medicine (Baltimore). 2023 Nov 24;102(47):e36283. doi: 10.1097/MD.0000000000036283 (PMC10681598; doi:10.1097/MD.0000000000036283)

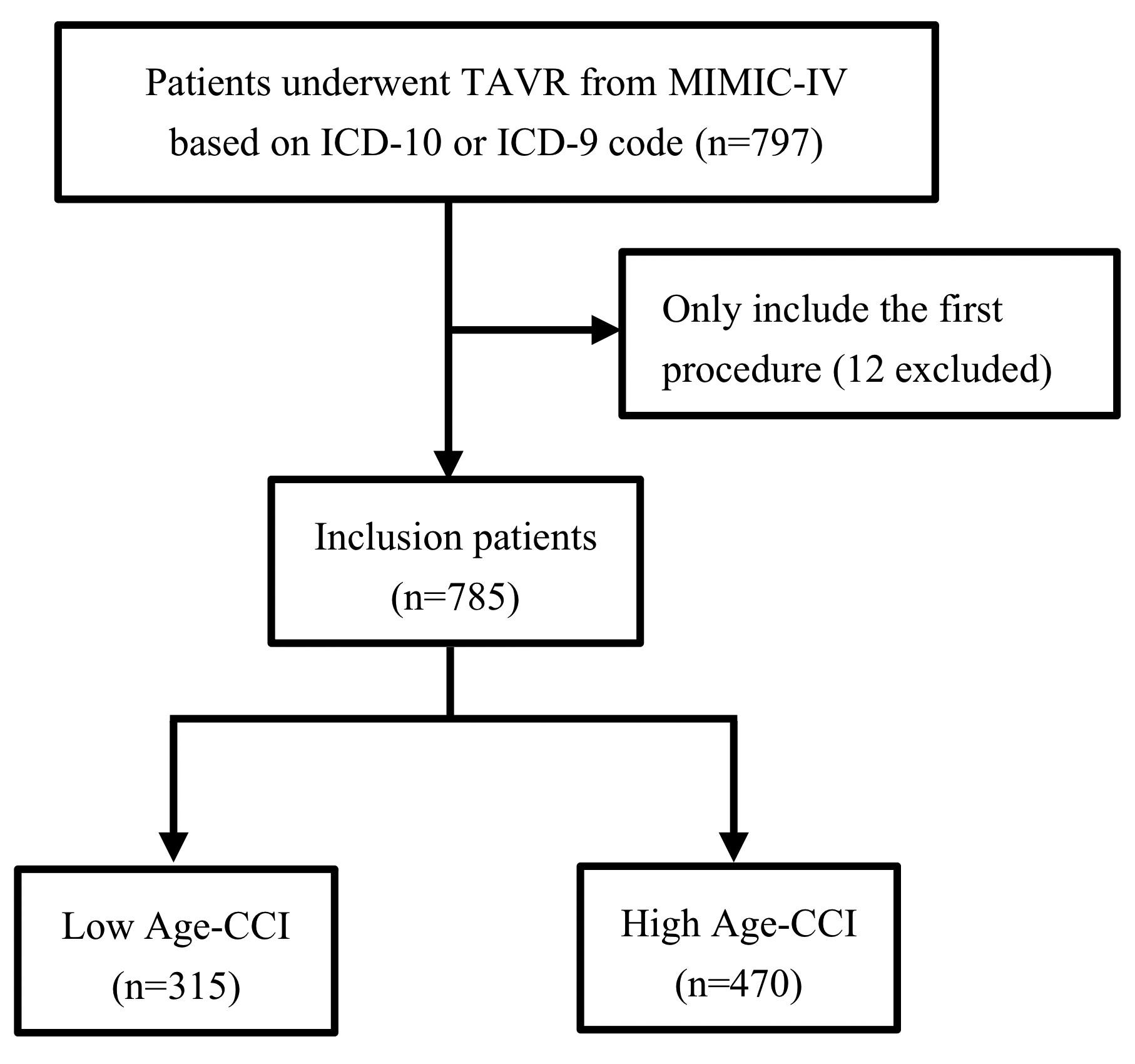

Supplement: Supplementary file 2 [file medi-102-e36283-s002.tif]

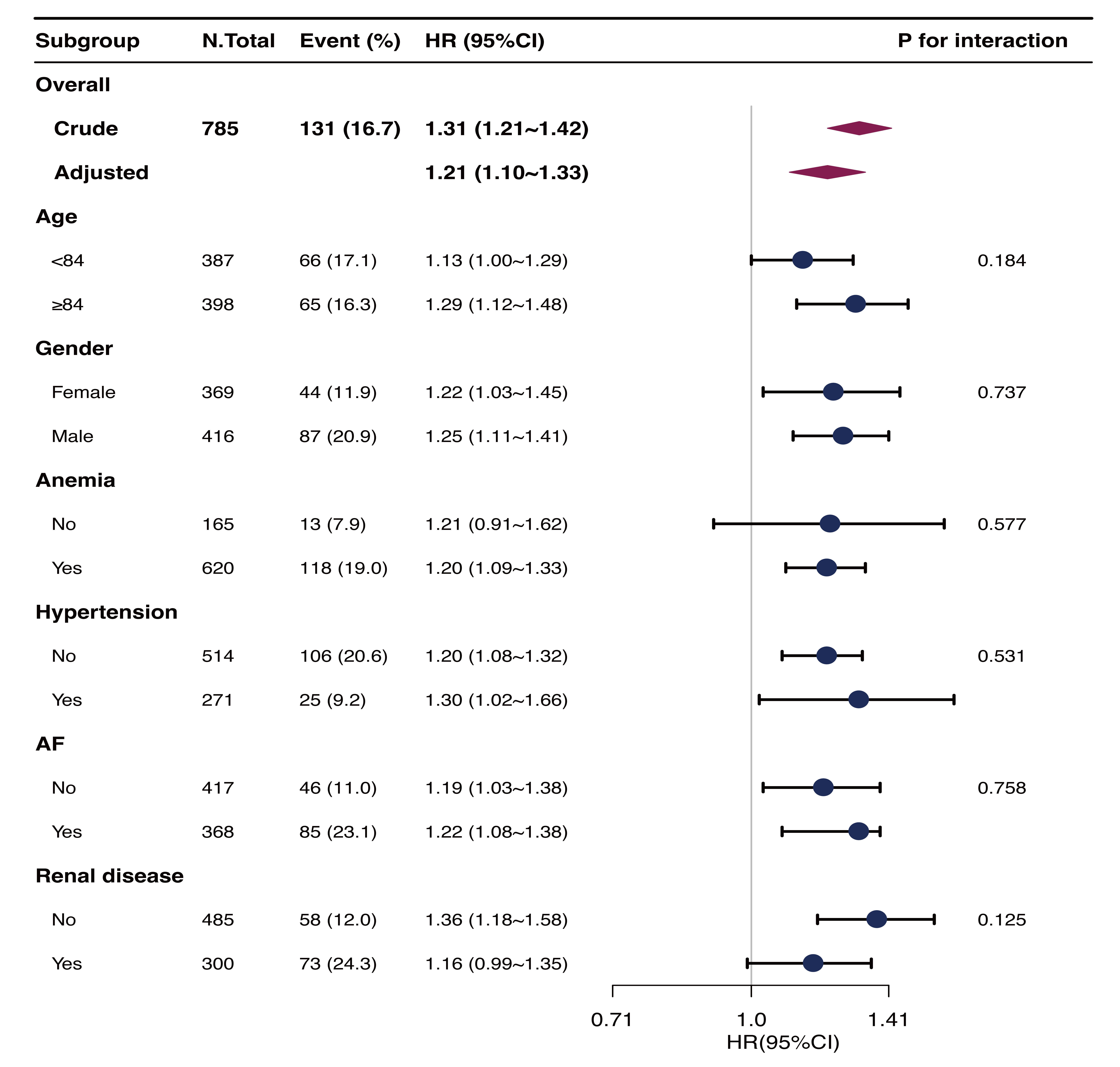

Supplement: Supplementary file 3 [file medi-102-e36283-s003.tif]
